# A New Genus of Tachydromiinae from South Africa and Turkmenistan (Diptera: Empidoidea: Hybotidae) †

**DOI:** 10.3390/insects13020171

**Published:** 2022-02-05

**Authors:** Bradley J. Sinclair, Jeffrey M. Cumming, Igor V. Shamshev

**Affiliations:** 1 Canadian National Collection of Insects and Ottawa Plant Laboratory-Entomology, CFIA, K.W. Neatby Bldg, C.E.F., 960 Carling Ave, Ottawa, ON K1A 0C6, Canada; 2 Canadian National Collection of Insects, Agriculture and Agri-Food Canada, K.W. Neatby Bldg, C.E.F., 960 Carling Ave, Ottawa, ON K1A 0C6, Canada; jeff.cumming@agr.gc.ca; 3 Laboratory of Insect Systematics, Zoological Institute, Russian Academy of Sciences, Universitetskaya Nab. 1, 199034 St. Petersburg, Russia; shamshev@mail.ru; 4 Laboratory of Agricultural Entomology, All-Russian Institute of Plant Protection, Podbelskogo Rd. 3, 188608 St. Petersburg, Russia

**Keywords:** Drapetidini, new species, disjunct distribution

## Abstract

**Simple Summary:**

A new hybotid dance fly (or fast-running fly) genus, *Parallelodromia* is described along with three new species. The new genus is recorded from South Africa and Turkmenistan. Details are provided regarding the morphology of the flies and the wide geographical gap between the species is discussed.

**Abstract:**

*Parallelodromia* gen. nov. is described with the inclusion of two new species from South Africa (*P*. *glenlyonensis* sp. nov. and *P*. *hantamica* sp. nov.) and a new species (*P*. *turkmenistanica* sp. nov.) from Turkmenistan. The genus is fully illustrated along with a discussion of its unusual disjunct geographic distribution and its phylogenetic relationship within the Tachydromiinae tribe Drapetidini.

## 1. Introduction

In their treatment of the Afrotropical Hybotidae, Sinclair and Cumming [1] referred to a distinctive new genus as “Undescribed genus B” based at the time on one undescribed South African species. Subsequently, a second South African species has been recognized and the third author discovered another new species from Turkmenistan in Middle Asia, which appeared to be congeneric with the two South African species. The purpose of this paper is to describe this new genus along with its three newly included species. The new genus belongs to the Tachydromiinae tribe Drapetidini (corrected spelling of Drapetini, see Sabrosky [2]) as characterized by Sinclair and Cumming [1,3] and Wahlberg and Johanson [4]. The phylogenetic relationship of the new genus to other Drapetidini genera is also briefly discussed along with its unusual disjunct geographic distribution.

The species of Drapetidini are small (1–2 mm) predatory flies that are generally seen running on leaves of vegetation, tree-trunks, stones, sandy biotopes, etc. [5,6]. The tribe currently comprises 19 genera, with about 550 species worldwide [7,8,9,10,11,12,13].

## 2. Materials and Methods

This study is based on material loaned from or deposited in the following institutions: Canadian National Collection of Insects, Ottawa, Canada (CNC); KwaZulu-Natal Museum, Pietermaritzburg, South Africa (NMSA); Zoological Institute, Russian Academy of Sciences, St. Petersburg, Russia (ZISP).

Terminology used for adult structures follow those of Cumming and Wood [14]. Label data for primary types are presented exactly as they appear. Data are listed from the top downward on the staging pin, with data from each label enclosed in quotation marks; lines are delimited by a forward slash mark. Additional information is included in square brackets. The repository of each type is given in parentheses. Secondary type data are abridged and listed alphabetically. Photographs were taken with a Leica camera model DFC5400 using Leica Application Suite X or a Nikon SMZ 1500 stereomicroscope equipped with a Nikon D700 digital SLR camera. The distribution map was created with Simplemappr [15].

The South Africa specimens were rather bleached and generally in poor condition due to long storage in alcohol prior to critical-point drying. Consequently, description of leg chaetotaxy was not possible.

## 3. Taxonomy

### 3.1. Parallelodromia *gen. nov.*

urn:lsid:zoobank:org:act:C9418EDB-B9B6-47EE-86CE-353585730D19

Undescribed genus B: Sinclair and Cumming, 2017: 1238, 1244, 1248.

Type species: *Parallelodromia hantamica* sp. nov. 

**Recognition.** This new genus is distinguished from all other genera of Tachydromiinae by the anteriorly arched wing vein M_1_ that parallels vein R_4+5_, eyes without ommatrichia and separated on face, gena distinctly extended below eye, cell br shorter than cell bm at apex, vein CuA+CuP very faint, often apparently detached basally.

**Diagnosis.** Very small (1.1–1.3 mm) brown to brownish black and densely greyish pruinose flies (Figure 1A,C). Frons moderately broad just below ocellar triangle (slightly less than width of ocellar triangle), with sides converging to antennae, narrow just above antennae. Eyes narrow, without ommatrichia; facets not enlarged. Face nearly as broad as frons just below ocellar triangle; parallel-sided (Figure 1B). Vertical setae prominent, 1 pair rather moderately long, inclinate. Ocellar tubercle with 2 pairs of setae, subequally short, anterior pair inclinate, posterior pair lateroclinate. Antenna with pedicel bearing ventral subapical seta, shorter than width of segment; postpedicel short, ovate, with dorsoapical extension; arista-like stylus arising dorsoapically, long and slender. Gena distinctly extended below eye. Occiput broad laterally, somewhat inflated. Palpus fusiform, flattened, with subapical seta. Thorax usually largely densely pruinose, except for shiny katepisternum. Prosternum separated from proepisternum. Proepisternum with 1 short seta just above fore coxa and 1 similar seta on upper part. Postpronotal lobe weakly developed, with postpronotal seta prominent. Lateral mesonotal setae prominent; 2 notopleurals; 0–1 presutural supra-alar; 1 postsutural supra-alar; 1 postalar; 1 apical scutellar pair and 1 shorter subapical scutellar pair; acrostichal setulae biserial, arranged in regular parallel rows, rows divergent on prescutellar depression; dorsocentral setulae (including intra-alars) in 2 or more rows, separated from rows of acrostichals. Legs short, fore tibial gland present; fore femur slightly swollen; mid leg lacking secondary sexual characters in male; tibia with short preapical setae, hind tibia with 1 anteroventral seta; without apical projection. Wings hyaline with short basal costal seta (Figure 1D); Rs long, originating proximal to middle of cell bm or near middle of R_1_; R_1_ meeting costa near middle of wing; R_2+3_ moderately long, meeting costa beyond mid-point of wing; vein M_1_ anteriorly arched, running parallel with vein R_4+5_, terminating anterior to wing tip; CuA+CuP present as fold, often apparently detached basally; crossvein CuA_2_ absent; crossvein bm-cu oblique; cell br shorter than cell bm at apex; cell bm broader than cell br. Abdomen with tergites 1–7 unmodified; squamiform setae and gland-like intersegmental structures absent. Male terminalia with epandrium completely divided; left epandrial lamella fused to hypandrium; left surstylus differentiated from epandrial lamella, single or paired; right surstylus not differentiated, right epandrial lamella broad and elongate; rod-shaped bacilliform process arising beneath left surstylus; cerci separated, with expanded margins around anus, left cercus enlarged and species specifically modified; hypoproct unmodified; hypandrium with some setae subapically; phallus long and filamentous (South African species) or short, rather subtubular, well sclerotized (Turkmenistan species); two rod-shaped apodemes (i.e., ejaculatory and ventral apodemes) present, ventral apodeme longer. Female similar to male, except frons slightly broader; segments 7 and 8 greatly reduced and partially retracted into segment 6; terminalia short; tergite 8 separated from sternite 8; syntergite 9+10 divided into pair of small lateral sclerites; cercus rather short, ovate.

**Etymology.** The genus epitaph is derived from the parallel state of the longitudinal veins M_1_ and R_4+5_, and –dromia is a commonly used suffix in Empidoidea, in reference to the running habits of the adults of many genera. The genus name is feminine.

### 3.2. Key to Species of Parallelodromia

**Figure 2:**
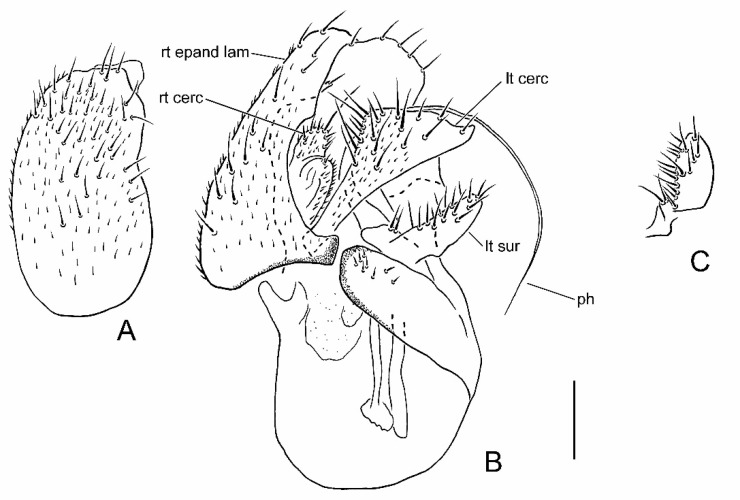
Male terminalia of *Parallelodromia glenlyonensis* sp. nov.: (**A**) right epandrial lamella, lateral view; (**B**) terminalia, dorsal view; (**C**) left surstylus, lateral view. Scale bar = 0.1 mm. Abbreviations: lt cerc—left cercus; lt sur—left surstylus; ph—phallus; rt cerc—right cercus; rt epand lam—right epandrial lamella.

**Figure 3:**
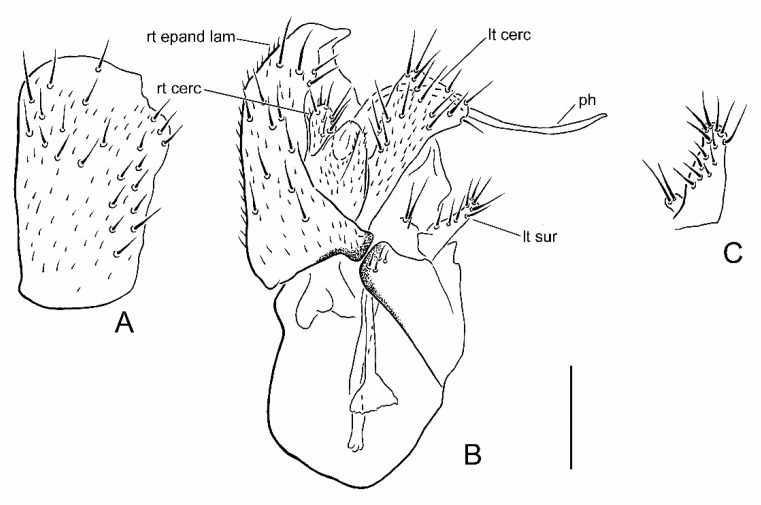
Male terminalia of *Parallelodromia hantamica* sp. nov.: (**A**) right epandrial lamella, lateral view; (**B**) terminalia, dorsal view; (**C**) left surstylus, lateral view. Scale bar = 0.1 mm. Abbreviations: lt cerc—left cercus; lt sur—left surstylus; ph—phallus; rt cerc—right cercus; rt epand lam—right epandrial lamella.

**Figure 1:**
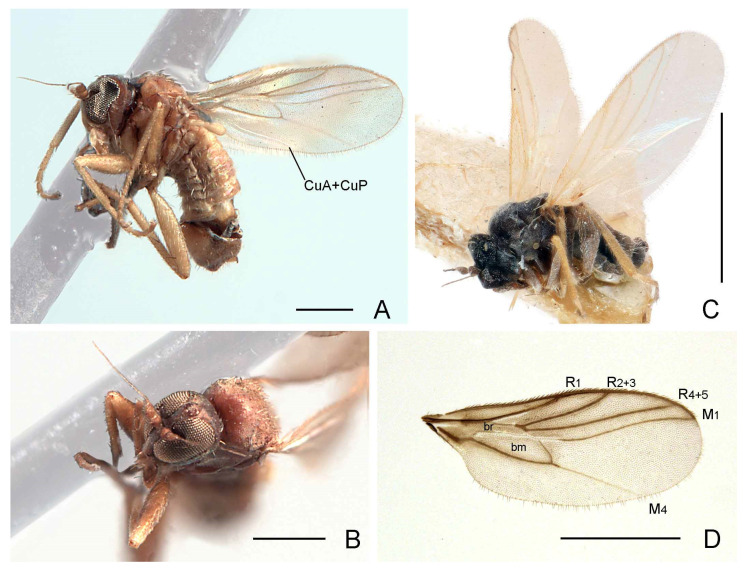
Species of *Parallelodromia* gen. nov.: (**A**) *P. glenlyonensis* sp. nov., male habitus, lateral view, scale bar = 0.25 mm; (**B**) *P. hantamica* sp. nov., head, oblique anterodorsal view, scale bar = 0.25 mm; (**C**) *P. turkmenistanica* sp. nov., male habitus, lateral view, scale bar = 1.0 mm; (**D**) *P. glenlyonensis* sp. nov., wing, scale bar = 0.5 mm. Abbreviations: bm—basal medial cell; br—basal radial cell; CuA+CuP—anterior branch of cubital vein + posterior branch of cubital view; M_1_—first branch of media; M_4_—fourth branch of media; R_1_—first branch of radius; R_2+3_—second branch of radius; R_4+5_—third branch of radius.

1. Setation of scutum pale; tibiae and basitarsi yellow (Figure 1C).......................................................***P. turkmenistanica* sp. nov.**

- Setation of scutum golden; tibiae and basitarsi pale brown to brown (Figure 1A).......................................................2

2. Halter knob brownish (Figure 1A); right epandrial lamella broad, somewhat pointed apically (Figure 2A); male left cercus triangular with prolonged outer corner (Figure 2B).......................................................***P. glenlyonensis* sp. nov.**

- Halter knob whitish; right epandrial lamella broad, rounded and truncate apically (Figure 3A); male left cercus triangular without prolonged outer corner (Figure 3B).......................................................***P. hantamica* sp. nov.**

### 3.3. Parallelodromia glenlyonensis *sp. nov.*

**Figure 4:**
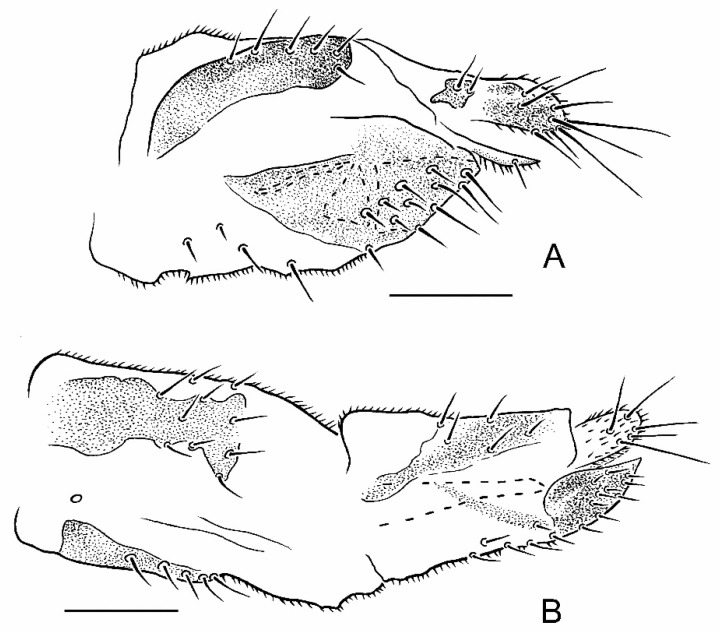
Female terminalia of *Parallelodromia*, lateral view: (**A**) *P. glenlyonensis* sp. nov.; (**B**) *P. hantamica* sp. nov., including segment 7. Scale bar = 0.1 mm.

(Figure 1A,D, Figure 2A–C, Figure 4A and Figure 6)

urn:lsid:zoobank:org:act:15E55395-78A8-429F-917B-2210BCC379F3

**Type material. HOLOTYPE** male, labelled: “SOUTH AFRICA: [Northern Cape] Farm/Glenlyon/Camel Koppie/S31.41324° E19.15802° [31°24′48″ S, 19°9′29″ E]/781m, 3–15.x.2005, MT/J. Schmidt”; “HOLOTYPE/Parallelodromia/glenlyonensis/Sinclair, Cumming, Shamshev” (NMSA). **PARATYPES:** Same data as holotype (8 males, 4 females, CNC; 1 male, 1 female, NMSA); same data as holotype except, 15–24.x.2005 (4 males, CNC); same data except, 15.ix.–3.x.2005 (3 females, CNC); same data as holotype except, 24.x.–3.xi.2005 (3 males, 4 females, CNC).

**Recognition.** Head, thorax and male terminalia dark brown (Figure 1A); abdomen and legs pale brown; halter knob brownish; right epandrial lamella (=right surstylus) broad and somewhat pointed apically; male left cercus triangular with prolonged outer corner.

**Description. Male.** Length. Body 1.1–1.3 mm; wing 1.0–1.1 mm. *Head*. Dark brown in ground-colour with whitish pruinescence; eyes brownish; setation brown. Frons linear, narrower just above antennae, moderately broad (slightly narrower than ocellar triangle) on upper part. Face linear, subequal to width of frons above antennae. Antenna uniformly brown; pedicel with ventral seta subequal to width of segment; postpedicel ovate, slightly longer than pedicel, with short, slender dorsoapical extension; arista-like stylus dorsoapical, about 3 times longer than postpedicel, pedicel and scape combined. Palpus broad, pale brown to yellowish brown, fusiform with slightly shorter pale subapical seta and some setulae. *Thorax*. Brown in ground-colour, paler than head, clothed in whitish pruinescence; mesonotum without pattern; setation brownish; katepisternum largely shiny, without pruinescence. *Legs*. Uniformly pale brown to yellowish brown. Femora evenly thickened; most outstanding setae broken off. *Wing* (Figure 1D). As in genus diagnosis; faintly infuscate, with largely brown veins; M_4_ pale; CuA+CuP faintly visible at certain angles. Halter with brownish knob. *Abdomen*. Pale brown, with short brown setation. *Terminalia* (Figure 2A–C). Rather broad in left lateral view, brown, largely subshiny. Cerci with unmodified setation; right cercus short, lobe-like; left cercus triangular more than twice as long, with prolonged outer corner. Epandrium completely divided. Right epandrial lamella (=right surstylus) enlarged, pruinescent, expanded apically and laterally with apex somewhat pointed. Left epandrial lamella fused with hypandrium, bearing several short setae. Left surstylus consisting of one large setose lobe and small protuberance, with several short, unmodified setae. Narrow rod-shaped bacilliform process arising beneath left surstylus. Phallus long and filamentous. Two rod-shaped apodemes present: upper ejaculatory apodeme with expanded apex. **Female.** Similar to male, except as follows: tergite 8 with deep, broad V-shaped invagination anteriorly; sternite 8 with posterior half more thickly sclerotized, subapical setae with pronounced sockets; syntergite 9+10 broadly divided into pair of small lateral sclerites, bearing pair of setae (Figure 4A). 

**Etymology.** Named after Glenlyon Farm, an important type locality for various species of insects and vascular plants.

**Distribution.** Afrotropical Region: South Africa, Northern Cape Province (Figure 6).

**Remarks.** The Glenlyon Farm, now the Hantam National Botanic Garden (near Nieuwoudtville) is in the winter rainfall region in Western Karoo. The type series was collected in the same Malaise trap as *Stuckenbergomyia* sp. A [16], a rare hybotid genus endemic to southern Africa.

### 3.4. Parallelodromia hantamica *sp. nov.*

(Figure 1B, Figure 3A–C, Figure 4B and Figure 6)

urn:lsid:zoobank:org:act:9DC1C1C0-8FDA-4BA8-A5B5-5B4A97B2AF40

**Type material. HOLOTYPE** male, labelled: “SOUTH AFRICA: [Northern Cape] Farm/Glenlyon/Camel Koppie/S31.41324° E19.15802° [31°24′48″ S, 19°9′29″ E],/781m, 3–15.x.2005, MT,/J. Schmidt”; “HOLOTYPE/Parallelodromia/hantamica/Sinclair, Cumming, Shamshev” (NMSA). **PARATYPES:** Same data as holotype (19 males, 12 females, CNC; 2 males, 2 females, NMSA); same data as holotype except, 15.ix.–3.x.2005 (1 male, 1 female, CNC); same data as holotype except, 15–24.x.2005 (7 males, CNC); same data as holotype except, 24.x.–3.xi.2005 (20 males, 19 females, CNC).

**Recognition.** Head, thorax and male terminalia brown; abdomen and legs pale brown to brown; halter knob whitish; right epandrial lamella (=right surstylus) broad, rounded and truncate apically; male left cercus triangular without prolonged outer corner.

**Description. Male.** Length. Body 1.2–1.5 mm; wing 1.1–1.3 mm. *Head*. Dark brown in ground-colour with whitish pruinescence; eyes brownish; setation brown. Frons linear, narrower just above antennae, moderately broad (slightly narrower than ocellar triangle) on upper part. Face linear, subequal to width of frons above antennae. Antenna uniformly brown; pedicel with ventral seta subequal to width of segment; postpedicel ovate, slightly longer than pedicel, with short, slender dorsoapical extension; arista-like stylus dorsoapical, about 3 times longer than postpedicel, pedicel and scape combined. Palpus broad, pale brown to yellowish brown, fusiform with slightly shorter pale subapical seta and some setulae. *Thorax*. Brown in ground-colour, paler than head, clothed in whitish pruinescence; mesonotum without pattern; setation brownish; katepisternum largely shiny, without pruinescence. *Legs*. Uniformly pale brown to yellowish brown. Femora evenly thickened; most outstanding setae broken off. *Wing*. As in genus diagnosis; faintly infuscate, with largely brown veins; M_3_ pale; CuA+CuP faintly visible at certain angles. Halter with whitish knob. *Abdomen*. Pale brown, with short brown setation. *Terminalia* (Figure 3A–C). Less prominent in left lateral view than *P. glenlyonensis* sp. nov., brown, largely subshiny. Cerci with unmodified setation; right cercus short, lobe-like; left cercus triangular more than twice as long, without prolonged outer corner. Epandrium completely divided. Right epandrial lamella (=right surstylus) enlarged, pruinescent, expanded apically and laterally with apex rounded, truncate. Left epandrial lamella fused with hypandrium, bearing several short setae. Left surstylus consisting of one large setose lobe and small protuberance, with several short, unmodified setae. Narrow rod-shaped bacilliform process arising beneath left surstylus. Phallus long and filamentous. Two rod-shaped apodemes present: upper ejaculatory apodeme with expanded apex. **Female.** Similar to male, except as follows: tergite 8 with broad U-shaped invagination anteriorly; sternite 8 without posterior half more thickly sclerotized, subapical setae without pronounced sockets; syntergite 9+10 broadly divided into pair of small lateral sclerites, bearing pair of setae (Figure 4B). 

**Etymology.** A name pertaining to Hantam Karoo, a region renowned for its endemic vascular plant flora.

**Distribution.** Afrotropical Region: South Africa, Northern Cape Province (Figure 6).

**Remarks.** See under *P. glenlyonensis* sp. nov.

### 3.5. Parallelodromia turkmenistanica *sp. nov.*

**Figure 5:**
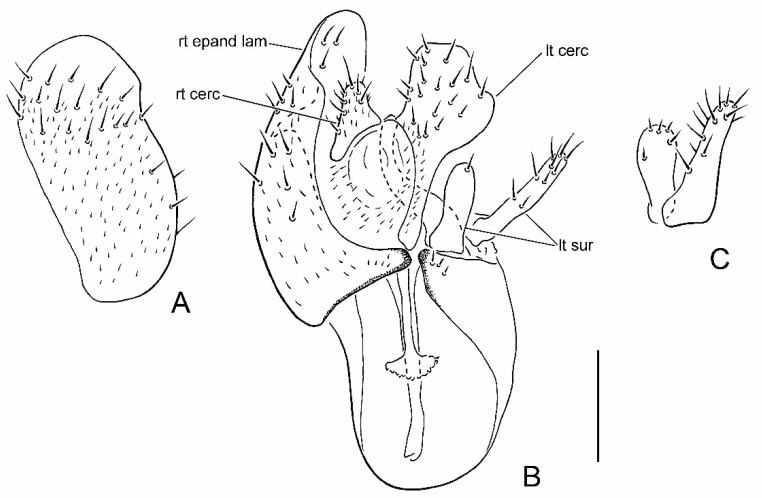
Male terminalia of *Parallelodromia turkmenistanica* sp. nov.: (**A**) right epandrial lamella, lateral view; (**B**) terminalia, dorsal view; (**C**) left surstylus, lateral view. Scale bar = 0.1 mm. Abbreviations: lt cerc—left cercus; lt sur—left surstylus; rt cerc—right cercus; rt epand lam—right epandrial lamella.

**Figure 6:**
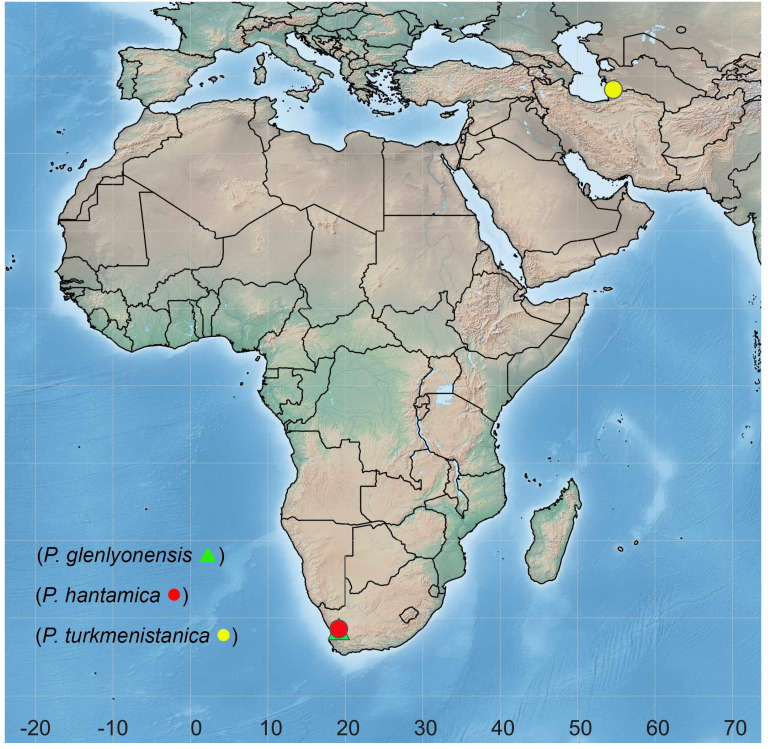
Distribution of *Parallelodromia*.

(Figure 1C, Figure 5A–C and Figure 6)

urn:lsid:zoobank:org:act:F058970F-74E0-4797-91DE-72F4D8B188DF

**Type material. Holotype** male labelled: “[printed in Cyrillic, Russian] Messerian [=Dahistan/Mishrian, 38°16’ N, 54°37´ E], otr. [=otrog, spur] Ko-/pet-dag [=Kopet Dag or Kopet Dagh], Turkm. [=Turkmenia, now Turkmenistan]/Steinberg 3.v.[1]951”; “Holotype/Parallelodromia/turkmenistanica/Sinclair, Cumming, Shamshev” (ZISP). **Paratype:** Same data as holotype (1 male, ZISP; dissected).

**Recognition.** Body only pale setose (except some setae on legs) (Figure 1C); head, thorax and male terminalia brown, abdomen pale brown, greyish pruinose; legs with femora almost entirely brownish (narrowly yellowish apically), tibiae entirely yellow, tarsomeres with basitarsi extensively yellow (only apex darkened), tarsomeres 2–5 brownish, becoming gradually darker to tarsomere 5; halter yellow.

**Description. Male.** Length. Body 1.2 mm; wing 1.1 mm (holotype). *Head*. Dark brown in ground-colour with greyish pruinescence; eyes brownish; setation pale. Frons triangular, very narrow just above antennae, moderately broad (slightly narrower than ocellar triangle) on upper part. Face parallel-sided, nearly as broad as frons just below ocellar triangle. Ocellar tubercle with 2 pairs of subequally short setae, anterior pair inclinate, posterior pair lateroclinate. One pair of moderately long, inclinate vertical setae. Antenna uniformly brown; pedicel with ventral seta subequal to width of segment; postpedicel rather drop-like, short, 2.3 times longer than pedicel, 1.4 times longer than basal width, with short, slender dorsoapical extension; arista-like stylus dorsoapical, 2.7 times longer than postpedicel, nearly as long as postpedicel, pedicel and scape combined. Palpus brown, fusiform, with slightly shorter pale subapical seta and some setulae. *Thorax*. Brown in ground colour, faintly greyish pruinose; mesoscutum without pattern; setation pale; katepisternum largely shiny, without pruinescence. Proepisternum with 1 short seta just above fore coxa and 1 similar seta on upper part. Postpronotal lobe with 1 moderately long seta. Mesonotal setae: presutural supra-alar space with 2–3 min setulae, 2 moderately long notopleurals (with some fine setulae anteriorly), 1 short postsutural supra-alar, 1 long postalar, 4 scutellars (apical setae very long, cruciate; lateral setae very short); acrostichals minute, arranged in 2 close, irregular rows, rather numerous, present on prescutellar depression; dorsocentrals separated from rows of acrostichals by broad bare space but undistinguishable from presutural intra-alar and supra-alar setulae, irregularly 2–3-serial anteriorly and becoming less numerous toward scutellum, mostly minute, prescutellar setae slightly longer. *Legs*. Fore coxa yellowish on apex, otherwise coxae brownish, faintly greyish pruinose; fore trochanter brownish yellow, mid and hind trochanters brownish; femora extensively brownish, narrowly yellowish apically (somewhat broader on fore femur); tibia entirely yellow; tarsomeres with basitarsi extensively yellow (only apex slightly darkened), tarsomeres 2–5 brownish, becoming gradually darker to tarsomere 5. Femora evenly thickened. Setation almost entirely pale, inconspicuous (except noted), minute. Fore coxa with scattered fine setae anteriorly (more numerous and longer closer to apex). Hind tibia with 1 dark, moderately long, anteroventral seta on about apical 1/4; without apical projection. *Wing*. As in genus diagnosis; faintly infuscate, with largely pale brown veins; M_3_ pale; CuA+CuP faintly visible at certain angles. Halter pale yellow. *Abdomen*. Pale brown in ground-colour, rather densely greyish pruinose, with short pale setation. *Terminalia* (Figure 5A–C). Rather moderately broad in left lateral view, dark brown, with pale setae. Cerci with unmodified setation; right cercus short, subtriangular (dorsal view), rounded apically; left cercus much larger than right cercus, rather subrectangular (dorsal view), with prolonged outer margin. Epandrium completely divided. Right epandrial lamella (=right surstylus) enlarged, pruinescent, elongate oval (lateral view), with broadly rounded apex; with some short simple setae. Left epandrial lamella fused with hypandrium, bearing 3 short setae. Left surstylus divided; upper lobe digitiform, elongate, narrow, with some short simple setae; lower lobe short, digitiform. Narrow rod-shaped bacilliform process arising beneath left surstylus. Hypandrium bare. Phallus short, rather subtubular, well sclerotized. Two rod-shaped apodemes present; upper ejaculatory apodeme with expanded apex; ventral apodeme longer, nearly reaching anterior margin of hypandrium (=gonocoxal apodeme). **Female.** Unknown.

**Etymology.** The epithet refers to the country of the origin of the new species.

**Distribution.** Palaearctic Region: Turkmenistan (Figure 6).

**Remarks.** The new species was collected from a spur of the northern part of Kopet Dag Range (or Turkmen-Khorasan Mountain Range), close to the Caspian Sea and the border between Turkmenistan and Iran. The type locality refers to the archaeological place in Turkmenistan (the former city Dahistan/Mishrian).

## 4. Discussion

Sinclair and Cumming [1] first recognized *Parallelodromia* gen. nov. as “Undescribed genus B” in the Tachydromiinae tribe Drapetidini based on a single species from South Africa. Two species (*P*. *glenlyonensis* sp. nov. and *P*. *hantamica* sp. nov.) are now recognized from the Northern Cape region of South Africa in addition to a single species (*P*. *turkmenistanica* sp. nov.) from Turkmenistan in Middle Asia. Despite this strange geographic distribution, the genus appears to be monophyletic based minimally on very distinctive wing venation with an anteriorly arched vein M_1_ that parallels vein R_4+5_ and eyes that lack ommatrichia, a reversal that is very rare in Drapetidini [3] (p. 21).

The relationships of *Parallelodromia* gen. nov. within Drapetidini are presently not evident. As discussed by Cumming [17], a short basal radial cell appears to be a synapomorphy of a clade of approximately 10 drapetidine genera, which would now also include *Parallelodromia* gen. nov. Cumming [17] suggested that this clade was made up of three lineages (*i.e.*, a *Drapetis* group of five genera, an *Austrodrapetis* group of three genera, and *Stilpon* Loew + *Baeodromia* Cumming), but that the relationships of these three lineages to each other was uncertain and required further study in particular, of male abdominal glands that occur in different forms in most taxa throughout the clade. Interestingly, *Parallelodromia* gen. nov. does not possess male abdominal gland-like modifications so its relationships within the short basal radial cell clade remain unclear if it actually belongs there.

The peculiar disjunct geographic distribution of *Parallelodromia* gen. nov. with two species in the Northern Cape region of South Africa and one species from Turkmenistan in Middle Asia (Figure 6) with no other species in between, is probably a function of available Mediterranean climate in this area and possibly also a collection artefact. This seems to be a rare biogeographic pattern that is known in a few groups of Diptera [18] and at least one other group of Empidoidea, the *Schistostoma albopilosum* group of Dolichopodidae, which only contains species in southern Africa and the Mediterranean region [19], plus one species from northern India [20].
